# Risks of complicated acute appendicitis in patients with psychiatric disorders

**DOI:** 10.1186/s12888-022-04428-7

**Published:** 2022-12-05

**Authors:** Junmo Kim, Chaeyoung Yang, Hyung Joon Joo, Rae Woong Park, Ga Eun Kim, Daeho Kim, Joonho Choi, Jun Ho Lee, Eunkyung Kim, Seon-Cheol Park, Kwangsoo Kim, Il Bin Kim

**Affiliations:** 1 Interdisciplinary Program in Bioengineering, Seoul National University, Seoul, Republic of Korea; 2 Department of Psychiatry, Hanyang University College of Medicine, Seoul, Republic of Korea; 3 Department of Psychiatry, Hanyang University Medical Center, Seoul, Republic of Korea; 4 Division of Cardiology, Department of Internal Medicine, Korea University Anam Hospital, Seoul, Republic of Korea; 5 Department of Medical Informatics, Korea University College of Medicine, Seoul, Republic of Korea; 6 Department of Biomedical Informatics, Ajou University School of Medicine, Suwon, Republic of Korea; 7 Department of Psychiatry, Ewha Womans University Mokdong Hospital, Ewha Womans University College of Medicine, Seoul, Republic of Korea; 8 Department of Psychiatry, Hanyang University Guri Hospital, Guri, Republic of Korea; 9 Department of Surgery, Hanyang University College of Medicine, Seoul, Republic of Korea; 10 Transdisciplinary Department of Medicine and Advanced Technology, Seoul National University Hospital, Seoul, Republic of Korea; 11 Department of Medicine, College of Medicine, Seoul National University, Seoul, Republic of Korea; 12 Graduate School of Medical Science and Engineering, Korea Advanced Institute of Science and Technology, Daejeon, Republic of Korea

**Keywords:** Psychiatric disorder, Complicated appendicitis, Common data model, Treatment compliance

## Abstract

**Background:**

Acute appendicitis often presents with vague abdominal pain, which fosters diagnostic challenges to clinicians regarding early detection and proper intervention. This is even more problematic with individuals with severe psychiatric disorders who have reduced sensitivity to pain due to long-term or excessive medication use or disturbed bodily sensation perceptions. This study aimed to determine whether psychiatric disorder, psychotropic prescription, and treatment compliance increase the risks of complicated acute appendicitis.

**Methods:**

The diagnosis records of acute appendicitis from four university hospitals in Korea were investigated from 2002 to 2020. A total of 47,500 acute appendicitis-affected participants were divided into groups with complicated and uncomplicated appendicitis to determine whether any of the groups had more cases of psychiatric disorder diagnoses. Further, the ratio of complicated compared to uncomplicated appendicitis in the mentally ill group was calculated regarding psychotropic dose, prescription duration, and treatment compliance.

**Results:**

After adjusting for age and sex, presence of psychotic disorder (odds ratio [OR]: 1.951; 95% confidence interval [CI]: 1.218–3.125), and bipolar disorder (OR: 2.323; 95% CI: 1.194–4.520) was associated with a higher risk of having complicated appendicitis compared with absence of psychiatric disorders. Patients who are taking high-daily-dose antipsychotics, regardless of prescription duration, show high complicated appendicitis risks; High-dose antipsychotics for < 1 year (OR: 1.896, 95% CI: 1.077–3.338), high-dose antipsychotics for 1–5 years (OR: 1.930, 95% CI: 1.144–3.256). Poor psychiatric outpatient compliance was associated with a high risk of complicated appendicitis (OR: 1.664, 95% CI: 1.014–2.732).

**Conclusions:**

This study revealed a close relationship in the possibility of complicated appendicitis in patients with severe psychiatric disorders, including psychotic and bipolar disorders. The effect on complicated appendicitis was more remarkable by the psychiatric disease entity itself than by psychotropic prescription patterns. Good treatment compliance and regular visit may reduce the morbidity of complicated appendicitis in patients with psychiatric disorders.

**Supplementary Information:**

The online version contains supplementary material available at 10.1186/s12888-022-04428-7.

## Background

Psychiatric disorders are generally recognized to increase the complication risk of physical disorders [1, 2]. Physical comorbidity is frequently under- or misdiagnosed in patients with psychiatric disorders, who may carry a risk of developing comorbid physical complications [3, 4]. A bodily sensation can be inappropriately expressed in a mixture of psychopathologic symptoms in patients with psychiatric disorders, which in turn delays an urgent surgical intervention for acute appendicitis [5]. Several studies indicated that patients with schizophrenia are prone to miss their chance of seeking timely therapeutic intervention after the onset of abdominal pain that is associated with appendicitis [6–8]. Meanwhile, other psychiatric disorders, such as affective disorders, rather than psychosis, have rarely been investigated for the risk of complicated acute appendicitis. Moreover, the risk of complicated acute appendicitis has little been studied from the perspective of medical factors, which possibly affect the course of physical comorbidity in patients with psychiatric disorders.

The risk of complicated acute appendicitis should be evaluated in terms of medical factors, including psychiatric disease entity, psychotropic prescription, and treatment compliance, because the medical factors affect the physical comorbidity course, as well as psychopathological symptoms, in the patients with psychiatric disorders [4]. First, a specific psychiatric disorder itself may predispose patients to experience an altered bodily sensation related to their physical comorbidity, thereby influencing a course of the comorbid disorders. Particularly, decreased pain sensitivity has been reported in studies of patients with psychotic disorders, such as schizophrenia [9, 10]. Patients with bipolar disorder also reported decreased pain sensitivity, especially in a manic phase [11, 12]. Meanwhile, studies have reported that other patients with depressive disorders have increased pain sensitivity [13–15]. Patients with significant medical events present without the usual signs of pain, resulting in misdiagnosis and delayed treatment. Indeed, patients with schizophrenia were found to have an increased risk of complicated acute appendicitis compared with controls [16, 17], whereas patients with depression have no increased complication risk [8]. Second, the psychotropic prescription may affect the pain sensitivity, thereby altering the course of the physical comorbidity. Previous studies have suggested that psychotropic medication may be responsible for altered pain sensitivity in patients with psychiatric disorders. The analgesic effect of the antipsychotics, such as haloperidol, has been proposed in patients with schizophrenia. Changes in pain response according to phenothiazine dosage have also been reported [18]. However, recent experimental studies have concluded that antipsychotic medications do not cause pain sensitivity changes [19], and thus far, consistent conclusions have not been reached. Various psychotropic medications, including antidepressants and anxiolytics, have also influenced the pain sensitivity of patients who take those medications in addition to antipsychotics [20, 21]. For instance, several studies suggest that pain and depressive symptoms both improve with tricyclic antidepressants, selective serotonin reuptake inhibitors, and serotonin and norepinephrine reuptake inhibitors [22, 23]. Anxiolytics, such as benzodiazepines, also significantly induce pain reduction [24]. Third, poor treatment compliance in patients with psychiatric disorders may affect the course of physical comorbidity, thus possibly increasing the risk of complicated acute appendicitis. Patients with psychiatric disorders are likely not to receive the necessary interventions for their physical symptoms due to altered bodily sensations. Physical symptom evaluation during outpatient psychiatry clinic visits can lead to early physical disease detection and thus appropriate treatment administration. Suboptimal treatment of the physical conditions in patients with schizophrenia has been several times suggested to contribute to their higher mortality. A review on hypertension concluded that one of the most common risk factors for cardiovascular morbidity was that patient with schizophrenia and bipolar disorder generally get poorer care, including lower screening rates and prescriptions compared with general populations [25]. The same conclusion was reached in another study, which showed that patient with schizophrenia had an increased risk of all-cause mortality, reinfarction, and stroke, despite a lower prevalence of traditional cardiac risk factors. Additionally, schizophrenia patients might have vulnerability to be underdiagnosed [26]. Considering those premises aforementioned, we set the comprehensive analytic approach that helps resolve various medical factors, including the psychiatric disorder entity, psychotropic prescription patterns, and treatment adherence, which might contribute to the risks of complicated acute appendicitis. Thus, we aimed to determine whether psychiatric disorder, psychotropic prescription, and treatment compliance increase the risks of complicated acute appendicitis. This study can enhance the attention of medical professionals, thereby prompting them not to miss the signs of urgent physical comorbidity in patients with psychiatric disorders.

## Methods

### Data curation

Observational Health Data Sciences and Informatics (OHDSI) is an international collaboration whose goal is to create and apply open-source data analytic solutions to an extensive network of health databases to improve human health and wellbeing [27]. OHDSI grew out of the Observational Medical Outcomes Partnership (OMOP), which is a public-private partnership established in the United States to inform the appropriate use of observational healthcare databases and study the effects of medical products. The OHDSI common data model (CDM) provides standard-based data analysis solutions that support converting electronic health records (EHR) from different sources into a standard data structure, which enables large-scale data analysis. The present study was performed using data from Korea’s four large medical centers (Seoul National University Hospital, Korea University Anam Hospital, Ajou University Medical Center, and Ewha Woman’s University Seoul Hospital) among 10 hospitals with OMOP-CDM in Korea [28]. The Institutional Review Board (IRB) at Seoul National University Hospital granted a waiver of approval and informed consent, considering that the data for this study (IRB No. 2102–064-1196) were de-identified and based on observational electronic medical records from the OHDSI research network. This retrospective observational cohort study was conducted according to the principles of the Declaration of Helsinki.

### Cohort definition and Main outcomes

Participants with acute appendicitis were collected from the OMOP-CDM datasets of the four general hospitals in Korea from January 2000 to December 2020. In this study, the diagnoses of acute appendicitis accompanied by peritonitis, perforation, or appendix abscess were defined as complicated acute appendicitis, and otherwise as uncomplicated acute appendicitis. A total of 47,500 people were enrolled (The total number of subjects was 47,500, wherein 9900 from Korea University Anam Hospital: 5800 from Seoul National University Hospital: 15,500 from Ewha Woman’s University Mokdong Hospital; and 16,300 from Ajou University Hospital). The whole participants were categorized according to the psychiatric disorder diagnosis and psychotropic medication prescription. First, we categorized the whole participants with or without psychiatric disorder diagnosis during the study period. Psychiatric disorders were classified based on the International Classification of Diseases-10 codes, and the details are shown in Supplementary Table 1 [29]. Participants without any psychiatric disorder diagnosis made within 5 years before the first diagnosis of acute appendicitis were excluded. Second, the whole participants were categorized into those with and without prescribed psychotropic medications. Participants with prescribed psychotropic medications were further stratified by the prescription dosage (i.e., low and high dose) and treatment compliance (i.e., good and poor compliance). The prescription dosage was defined as the total dose of psychotropic medications divided by the defined daily dose (DDD) [30] and the prescribed number of days. A low dose was defined as < 1 and a high dose as ≥1. Here, we exploited information on anatomical therapeutic chemical (ATC) codes and DDD for selected psychotropic medications, including antipsychotics (ATC code N05A), antidepressants (ATC code N06A), and anxiolytics (ATC codes N05B and N05C) [31]. Treatment compliance was defined as the ratio of the actual prescription duration divided by the total follow-up duration from the first to the last prescription. The cut-off ratio of high and low compliance was set at 0.8. For all of the aforementioned analyses, the outcome of interest was the presence of complicated acute appendicitis during the follow-up.

### Statistical analysis

Differences in demographic characteristics, including age, sex, and ethnicity, were measured between the acute appendicitis-affected participants with complicated and uncomplicated acute appendicitis, using the χ2 test and standardized difference. The logistic regression model was used to estimate a relative hazard of the complicated acute appendicitis, in which the odds ratio (OR) was adjusted by age and sex. The overall effects were evaluated using the meta-analysis approach, which combined the results of the four general hospitals in Korea. We evaluated the heterogeneity among participants of the different cohorts by calculating the I^2^ value. We used each of the analysis results selectively according to the I^2^ value assigned to the meta-analysis results for the cohorts. A fixed-effect model was used if the I^2^ value was < 50. Otherwise, a random-effect model was used. A two-sided *p*-value of < 0.05 was considered statistically significant. All analyses were performed using R version 4.1.0 from the Free Software Foundation, Inc. (http://cran.r-project.org).

## Results

### Participant demographics

Table 1 shows the characteristics of the study samples according to the presence of complicated acute appendicitis. Several patients were 14 years or younger, followed by 25–34 years old, and 15–24 years old. Regarding psychiatric disorder diagnosis, 98.25% of the participants had no disorders, 0.78% had depressive disorders, and 0.54% had anxiety disorders. The ratio of males and over-middle-aged was higher in the complicated appendicitis group than in the uncomplicated appendicitis group. The distribution of psychiatric disorders in those with complicated acute appendicitis was as follows: depressive disorder (40.6%, 86/212), psychotic disorder (13.2%, 28/212), and bipolar disorder (6.60%, 14/212). The distribution of psychiatric disorders in those with uncomplicated appendicitis was as follows: depressive disorder (34.6%, 286/827), psychotic disorder (7.38%, 61/827), and bipolar disorder (3.99%, 33/827). Participant demographics for each hospital are shown in Supplementary Tables 2, 3, 4, and 5.

**Table 1:** Participant characteristics of complicated and uncomplicated acute appendicitis

	Complicated Appendicitis (*n* = 8158)	Uncomplicated Appendicitis (*n* = 39,360)	Total (*n* = 47,518)	Standardized difference
	n (%)	n (%)	n (%)	
**Sex**				
Men	4300 (52.71)	19,850 (50.43)	24,150 (50.82)	0.021
**Age**				
≤ 14	1442 (17.68)	8486 (21.56)	9928 (20.89)	0.073
15–24	942 (11.55)	7195 (18.28)	8137 (17.12)	0.153
25–34	1142 (14.00)	8420 (21.39)	9562 (20.12)	0.150
35–44	1255 (15.38)	5991 (15.22)	7246 (15.25)	0.004
45–54	1037 (12.71)	3914 (9.94)	4951 (10.42)	0.074
55–64	954 (11.69)	2668 (6.78)	3622 (7.62)	0.149
65–74	739 (9.06)	1665 (4.23)	2404 (5.06)	0.177
≥ 75	634 (7.77)	989 (2.51)	1623 (3.42)	0.223
**Ethnicity**				
Korean	8028 (98.41)	38,904 (98.84)	46,932 (98.77)	0.002
**Psychiatric Diseases**				
No Disorders	7972 (97.72)	38,716 (98.36)	46,688 (98.25)	0.003
Anxiety Disorder	49 (0.60)	208 (0.53)	257 (0.54)	0.010
Obsessive Disorder	4 (0.05)	12 (0.03)	16 (0.00)	0.009
Trauma and Stress	27 (0.33)	103 (0.26)	130 (0.27)	0.013
Psychotic Disorder	28 (0.34)	61 (0.15)	89 (0.19)	0.038
Bipolar Disorder	14 (0.17)	33 (0.08)	47 (0.10)	0.025
Depressive Disorder	86 (1.05)	286 (0.73)	372 (0.78)	0.034
Personality Disorder	0 (0.00)	12 (0.03)	12 (0.03)	0.004
Dissociative Disorder	3 (0.04)	24 (0.06)	27 (0.06)	0.011
Somatoform Disorder	21 (0.26)	88 (0.22)	109 (0.23)	0.007

### Risks of complicated appendicitis according to psychiatric disorder entities

**Fig. 1:**
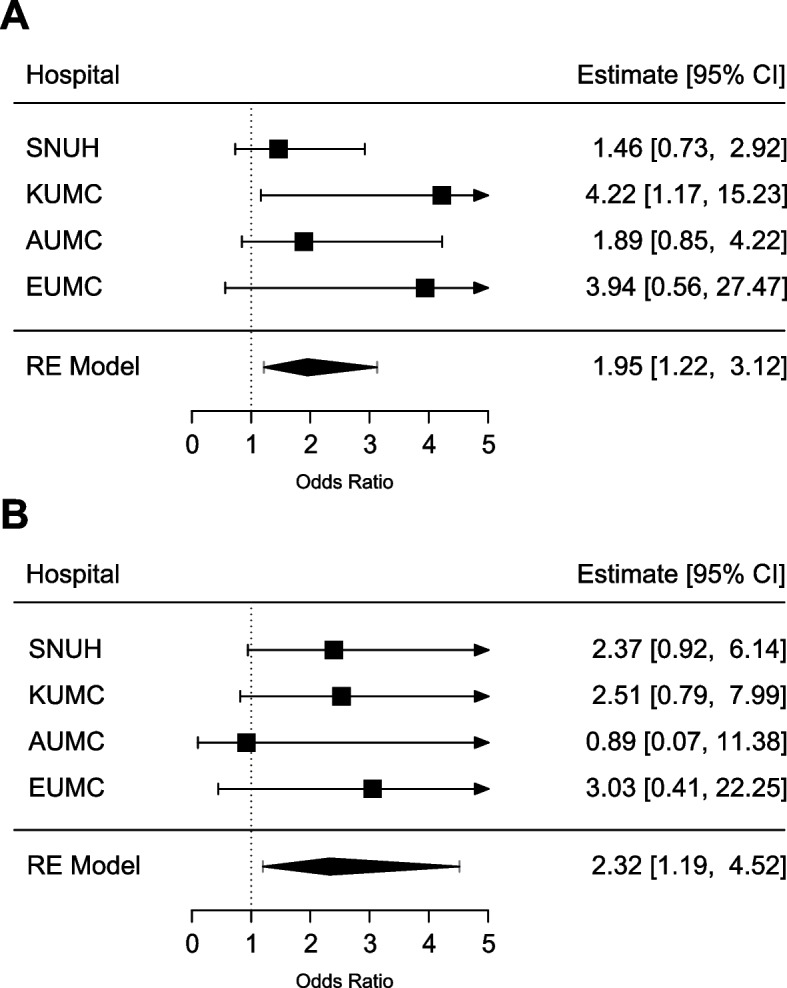
Risks of complicated acute apependicitis in psychiatric patients. **A**: psychotic disorder, **B**: bipolar disorder. SNUH: Seoul National University Hospital; KUMC: Korea University Anam Hospital; AUMC: Ajou University Medical Center; EUMC: Ewha Woman’s University Seoul Hospital; RE: random effect; CI: confidence interval

Table 2 shows the relative hazard for complicated acute appendicitis. The simple logistic regression analysis revealed that patients with psychotic disorder (OR: 2.362, 95% CI: 1.384–4.030) and bipolar disorder (OR: 2.627, 95% CI: 1.364–5.059) had significantly higher complication rates than patients with acute appendicitis without psychiatric disorders. The rate of complicated acute appendicitis showed a tendency to be higher in the case of depressive disorder (OR: 1.615, 95% CI: 0.902–2.893) than that of the reference group, although it was not statistically significant. No statistically significant difference was found for other disorders. After adjusting for age and sex, the OR of complicated appendicitis slightly decreased but was still significantly higher in patients with bipolar disorder (OR: 1.951, 95% CI: 1.218–3.125) and patients with psychotic disorder (OR: 2.323, 95% CI: 1.194–4.520). Patients with depressive disorder (OR: 1.130, 95% CI: 0.703–1.816) remained insignificant regarding elevated complicated appendicitis rates after adjusting for age and sex. Figure 1 shows the results of meta-analysis with forest plots of adjusted ORs for psychotic disorder and bipolar disorder.

**Table 2:** Risks of complicated acute appendicitis^a^

	Unadjusted OR (95% CI)	Unadjusted I^2^	Adjusted OR (95% CI)	Adjusted I^2^
**Psychiatric Disorders**				
No Disorders	1		1	
Anxiety Disorder	1.132 (0.625–2.050)	66.786	0.819 (0.513–1.308)	45.726
Obsessive Disorder	1.915 (0.559–6.554)	0.000	2.016 (0.587–6.928)	0.000
Trauma and Stress	1.269 (0.822–1.959)	0.000	1.121 (0.722–1.739)	0.000
Psychotic Disorder	2.362 (1.384–4.030)	16.255	1.951 (1.218–3.125)	0.000
Bipolar Disorder	2.627 (1.364–5.059)	0.000	2.323 (1.194–4.520)	0.000
Depressive Disorder	1.615 (0.902–2.893)	82.125	1.130 (0.703–1.816)	71.874
Personality Disorder	NA	NA	NA	NA
Dissociative Disorder	0.532 (0.115–2.465)	0.000	0.416 (0.089–1.945)	0.000
Somatoform Disorder	1.135 (0.628–2.053)	22.650	0.826 (0.504–1.353)	0.000
**Sex**				
Female	1		1	
Male	1.056 (0.959–1.163)	71.692	1.102 (0.982–1.238)	79.891
**Age**				
≤ 14	1		1	
15–24	0.796 (0.433–1.462)	97.329	0.792 (0.422–1.483)	97.466
25–34	0.825 (0.423–1.610)	98.010	0.828 (0.414–1.657)	98.124
35–44	1.267 (0.667–2.405)	97.855	1.256 (0.652–2.419)	97.936
45–54	1.664 (0.900–3.077)	97.471	1.638 (0.881–3.044)	97.491
55–64	2.373 (1.184–4.754)	97.930	2.379 (1.168–4.849)	98.000
65–74	2.915 (1.634–5.201)	96.366	2.909 (1.618–5.232)	96.437
≥ 75	4.301 (2.390–7.741)	95.741	4.275 (2.323–7.868)	95.964

^a^Adjusted for age and sex

Other characteristics, such as old age, were also significantly associated with a higher risk of complicated appendicitis, similar to the previous studies [32].

### Risks of complicated appendicitis according to type and dose of psychotropic prescription

Table 3 shows the OR for complicated acute appendicitis according to the prescribed psychotropic drugs for patients. Overall, the risk of complicated acute appendicitis was higher in patients taking psychotropic medication than in those who do not. High-daily-dose antipsychotics increased the risk of complicated appendicitis when considering age and sex. Regardless of duration, the high-dose antipsychotic group showed almost twice the risk of complicated appendicitis compared to the no-medication group; high-dose antipsychotics for < 1 year (OR: 1.896, 95% CI: 1.077–3.338) and high-dose antipsychotics for 1–5 years (OR: 1.930, 95% CI: 1.144–3.256). The risk of complicated appendicitis with anxiolytics or antidepressants was not statistically significant when considering age and sex.

**Table 3:** Risks of complicated acute appendicitis according to psychotropic doses^a^

Psychotropics (Dose)		Unadjusted OR (95% CI)	Unadjusted I^2^	Adjusted OR (95% CI)	Adjusted I^2^
No medication		1		1	
**Antipsychotics**					
for < 1Y	(low)	1.468 (1.012–2.129)	0.000	1.125 (0.771–1.640)	0.000
	(high)	3.240 (1.614–6.504)	89.662	1.896 (1.077–3.338)	83.278
for 1–5Y	(low)	5.767 (1.067–31.172)	0.000	2.538 (0.419–15.369)	0.000
	(high)	2.817 (1.679–4.725)	0.000	1.930 (1.144–3.256)	0.000
**Anxiolytics**					
for < 1Y	(low)	1.668 (1.342–2.074)	86.333	1.213 (0.959–1.535)	87.066
	(high)	1.909 (1.156–3.154)	71.497	1.363 (0.850–2.186)	66.618
for 1–5Y	(low)	2.092 (1.204–3.635)	64.321	1.197 (0.816–1.757)	23.800
	(high)	2.578 (0.737–9.021)	0.000	1.470 (0.418–5.170)	0.000
**Antidepressants**					
for <1Y	(low)	1.485 (1.153–1.914)	58.857	1.008 (0.788–1.289)	53.594
	(high)	1.429 (0.869–2.350)	63.000	1.142 (0.739–1.766)	50.683
for 1–5Y	(low)	2.382 (1.545–3.674)	28.056	1.329 (0.914–1.931)	1.400
	(high)	1.964 (1.269–3.039)	0.000	1.389 (0.893–2.160)	0.000

^a^Adjusted for age and sex. Dose = Total quantity / (DDD*Medication period), low for dose <1

### Risks of complicated appendicitis according to treatment compliance

Table 4 describes the risk of complicated acute appendicitis according to treatment compliance. Both the poor compliance antipsychotic group (compliance< 0.8) (OR: 1.664, 95% CI: 1.014–2.732) and good compliance antipsychotics group (compliance≥0.8) (OR: 1.437, 95% CI: 1.074–1.922) show increased risks of complicated appendicitis when age and sex were adjusted. The risks remained higher in the group that takes antipsychotics than that of the group not taking antipsychotics although the risks of complicated appendicitis decreased as compliance increased.

**Table 4:** Risks of complicated acute appendicitis according to treatment compliance^a^

Psychotropics (Dose)	Unadjusted OR (95% CI)	Unadjusted I^2^	Adjusted OR (95% CI)	Adjusted I^2^
**No medication**	1		1	
**Antipsychotics**				
Compliance < 0.8	2.831 (1.497–5.355)	79.301	1.664 (1.014–2.732)	64.241
Compliance ≥0.8	2.232 (1.468–3.394)	75.380	1.437 (1.074–1.922)	47.332
**Anxiolytics**				
Compliance < 0.8	2.004 (1.421–2.828)	90.313	1.299 (0.938–1.801)	88.059
Compliance ≥0.8	1.531 (1.274–1.840)	71.035	1.174 (0.974–1.414)	69.733
**Antidepressants**				
Compliance < 0.8	1.781 (1.136–2.793)	79.458	1.167 (0.816–1.668)	65.697
Compliance ≥0.8	1.550 (1.220–1.970)	57.587	1.068 (0.808–1.410)	66.616

^a^Adjusted by age and sex. Drug compliance = Actual duration of medication / Total duration of medication

## Discussion

This large-scale study systematically analyzed the risks of complicated acute appendicitis in patients with psychiatric disorders and revealed that potential medical factors, including the psychiatric disease entity, psychotropic prescription, and treatment compliance, can contribute to the increased risks of complicated appendicitis independent of age and sex. Psychiatric disorders, especially bipolar and psychotic disorders, increased the risk of complicated appendicitis. High-daily-dose antipsychotic users showed a high risk of complicated appendicitis. The higher the treatment compliance, the lower the risk of complicated appendicitis.

### Psychotic and bipolar disorders increase the risks of complicated appendicitis

Our findings show that certain psychiatric disorders are significantly associated with the increased risks of complicated acute appendicitis (psychotic disorder OR: 1.951 and bipolar disorder OR: 2.323). The delayed diagnosis of acute appendicitis may cause a higher complication rate due to the alteration of pain perception. Decreased pain sensitivity among patients with schizophrenia has been cited, with numerous examples of individuals with schizophrenia suffering from acutely painful medical conditions without reporting pain complaints [5, 9, 33–35]. Patients with significant medical events, such as myocardial infarctions, severe peptic ulcer disease, perforated bowel, peritonitis, femur fracture, and acute appendicitis present without the usual signs of pain, resulting in misdiagnosis, delayed treatment, and even death. Additionally, studies revealed that schizophrenia diagnosis is less frequent than expected in cohorts of patients with chronic pain, and the prevalence of pain complaints in schizophrenia is lower than among other psychiatric disorders [11, 12, 36–38]. Experimental studies generally described a higher pain threshold and tolerance in patients with schizophrenia than in healthy controls [9, 14, 39, 40]. The increased pain threshold even extends to the first degree relatives of patients with schizophrenia [41], supporting the strong inherent influence of the psychiatric disorder on altered pain sensitivity. Smilar to schizophrenia, bipolar disorder also appears to be associated with decreased pain sensitivity, although the evidence needs further corroboration. Some studies have investigated the relationship between patients with bipolar disorder and pain sensitivity, and even fewer have looked at pain responses in actively manic patients. Bipolar disorder was not widely reported in populations of patients with chronic pain, and complaints of pain are less frequently elicited in manic patients [11, 12]. One experimental study found decreased pain sensitivity among patients with bipolar in both manic and depressive states [40]. These studies indicate that decreased pain sensitivity in bipolar and psychotic disorders may have influenced appendicitis progression, although more investigation of the influence needs to be warranted.

### Patients with severe psychopathology taking high-dose antipsychotics show an increased risk of complicated appendicitis

Significantly increased complicated acute appendicitis were found in the high-daily-dose antipsychotic group. Concurrently, our result shows no considerable difference in the complication risks regarding the antipsychotic administration duration. Therefore, the disease itself may make a bigger contribution to the increased risk of complicated acute appendicitis rather than the prescription duration. This result is consistent with a previous experimental study that antipsychotic medications did not affect the patients’ pain threshold over a certain period. No significant change was found in the pain threshold that was measured after 8 weeks of medication wash-out and retaking antipsychotics for 3 days [19]. To our best knowledge, no studies have adequately explored the association between antipsychotic dose and subjective pain sensitivity.

Barbui et al. [42] noted that high antipsychotic doses were related to greater severity of psychopathology. High-dose antipsychotic use was associated with multiple admissions, positive psychotic symptoms, physical aggression, and more first-generation and less second-generation antipsychotics and antipsychotic polytherapy [43]. A previous experimental study revealed that the subjective pain threshold increases as the severity of psychotic symptoms increases in patients with schizophrenia. A strong negative correlation was found between negative symptoms and subjective pain threshold of artificial pain on lower extremities in patients with schizophrenic [44]. The higher severity of psychotic symptoms in high-dose antipsychotic users than the the medication itself would have had a more substantial effect on lowering pain sensitivity.

### Good treatment compliance decreases the risk of complicated appendicitis

The poorer the compliance with psychiatric treatment, the higher the risk of complicated acute appendicitis. This is in line with the results of previous studies that showed that the complication rate due to physical illness was high in the group with poor psychiatric treatment compliance [45]. However, even when regularly visiting the hospital, the prevalence of complicated appendicitis in patients with psychiatric disorders is higher than in general patients. This may be because the altered bodily sensation in patients with psychiatric disorders makes the recognition of their somatic symptoms challenging for both the patient and the practitioner. Clinically, nearly 40% of individuals with schizophrenia who suffer pain do not report it to their care provider [46]. Suboptimal treatments of medical conditions, such as hypertension and myocardial infarction, were reported several times [25, 26]. Likewise, inadequate treatment was also seen for case of breast cancer and females with schizophrenia had a 50% lower chance of receiving guideline treatment [47]. A similar pattern was seen in patients with chronic obstructive pulmonary disease, and persons with schizophrenia had a lower probability of following treatment guidelines [48].

The proper pain response may not be observed in psychiatric patients during physical examination, and accordingly a high close attention and examination are required in patients with psychiatric disorders. The attending physician’s physical examination (for discomfort, pain, and symptoms) of patients with psychiatric disorders will be the best way to promptly diagnose urgent physical comorbidity such as acute appendicitis and reduce the likelihood of complication transitions. Clinicians often focus only on psychopathologic symptoms when dealing with psychiatric disorders, which may partially lead to a delayed diagnosis of other physical comorbidities. Considering the reduced pain sensitivity of patients with psychiatric disorders, we should not neglect the examination of patients’ physical signs and symptoms, as well as psychiatric disease diagnosis. This is something to consider all the time, not only for psychiatrists but also for primary care practitioners who treat patients with a presumed psychiatric history.

Several study limitations should be noted. First, mood stabilizers that may affect pain sensitivity have not been investigated due to data collection limitations. Second, the mechanism for reduced pain sensitivity in specific psychiatric disorders has not been fully understood so far and thus not addressed in this paper: The pathophysiology linking pain and psychiatric disorders should be further studied in the future. Other possible medical factors affecting increased complicated acute appendicitis that were not covered in this study may include socioeconomic status, type of medical insurance, sociocultural aspect of treatment-seeking, psychiatric disease severity, comorbidity, and illness chronicity [49–53]. Further study is needed to find mediating factors between psychiatric disorders and complication risks with more detailed study cohort.

## Conclusions

This study revealed a close relationship between the risks of complicated appendicitis and psychiatric disorders, including psychotic and bipolar disorders. Additionally, the critical factor eliciting complicated acute appendicitis in psychiatric patients was the disease itself, but not the duration of taken medication. The medication dosage seemed to reflect the psychopathologic severity of patients. Briefly, the risks of complicated appendicitis in patients with psychiatric disorders is derived from the certain disease entity that might be plausibly associated with decreased pain sensitivity. We also addressed that good treatment compliance and regular visits can reduce the complicated acute appendicitis morbidity. Therefore, medical personnel should remain alert to the possibility that serious, potentially life-threatening physical conditions occur in patients with psychotic and bipolar disorders, who infrequently present with pain symptoms.

## Supplementary Information

**Additional file 1: Supplementary Table 1.** Psychiatric disorder diagnosis codes used for acute appendicitis-affected participants. **Supplementary Table 2.** Characteristics of acute appendicitis-affected participants with and without complications (SNUH). **Supplementary Table 3.** Characteristics of acute appendicitis-affected participants with and without complications (KUMC). **Supplementary Table 4.** Characteristics of acute appendicitis-affected participants with and without complications (AUMC). **Supplementary Table 5.** Characteristics of acute appendicitis-affected participants with and without complications (EUMC).

## Data Availability

The datasets generated during and/or analyzed during the current study are available from the corresponding author on reasonable request.

## Abbreviations

ATC: Anatomical therapeutic chemical
CDM: Common data model
CI: Confidence interval
DDD: Defined daily dose
EHR: Electronic health records
IRB: Institutional Review Board
OHDSI: Observational Health Data Sciences and Informatics
OMOP: Observational Medical Outcomes Partnership
OR: Odds ratio
