# Supplementary material for: Risks of complicated acute appendicitis in patients with psychiatric disorders
Source: BMC Psychiatry. 2022 Dec 5;22:763. doi: 10.1186/s12888-022-04428-7 (PMC9721022; doi:10.1186/s12888-022-04428-7)
Supplement: Supplementary file 1 — Additional file 1: Supplementary Table 1. Psychiatric disorder diagnosis codes used for acute appendicitis-affected participants. Supplementary Table 2. Characteristics of acute appendicitis-affected participants with and without complications (SNUH). Supplementary Table 3. Characteristics of acute appendicitis-affected participants with and without complications (KUMC). Supplementary Table 4. Characteristics of acute appendicitis-affected participants with and without complications (AUMC). Supplementary Table 5. Characteristics of acute appendicitis-affected participants with and without complications (EUMC). [file 12888_2022_4428_MOESM1_ESM.docx]

**Supplementary Table 1.** Psychiatric disorder diagnosis codes used for acute appendicitis-affected participants

| Psychiatric Disorders | ICD-10 Codes |
| --- | --- |
| Anxiety Disorder | F40-F41 and subcodes |
| Obsessive Disorder | F42 and subcodes |
| Trauma and Stress | F43 and subcodes |
| Psychotic Disorder | F20-F29 and subcodes, F33.3, F31.5, F30.2 |
| Bipolar Disorder | F30-F31 and subcodes |
| Depressive Disorder | F32-F39 and subcodes except F32.3, F33.3, F33.4 F34.0, F34.1, F34.9, F38.8, F39 |
| Personality Disorder | F60 and subcodes |
| Dissociative Disorder | F44 and subcodes |
| Somatoform Disorder | F45 and subcodes except F45.2 |

**Supplementary Table 2.** Characteristics of acute appendicitis-affected participants with and without complications (SNUH)

|  | With  Complication  (n=934) | Without Complication  (n=4,869) | Total  (n=5,803) | Standardized difference |
| --- | --- | --- | --- | --- |
|  | n (%) | n (%) | n (%) |  |
| **Sex** |  |  |  |  |
| Men | 470 (50.32) | 2,582 (53.03) | 3,052 (52.59) | 0.019 |
| **Age** |  |  |  |  |
| ≤ 14 | 209 (0.22) | 1,082 (0.22) | 1,291 (22.25) | 0.003 |
| 15ㅡ24 | 61 (0.07) | 698 (0.14) | 759 (13.08) | 0.249 |
| 25ㅡ34 | 86 (0.09) | 899 (0.18) | 985 (16.97) | 0.225 |
| 35ㅡ44 | 91 (0.1) | 578 (0.12) | 669 (11.53) | 0.07 |
| 45ㅡ54 | 108 (0.12) | 514 (0.11) | 622 (10.72) | 0.034 |
| 55ㅡ64 | 150 (0.16) | 509 (0.1) | 659 (11.36) | 0.169 |
| 65 – 74 | 130 (0.14) | 400 (0.08) | 530 (9.13) | 0.211 |
| ≥ 75 | 99 (0.11) | 189 (0.04) | 288 (4.96) | 0.43 |
| **Ethnicity** |  |  |  |  |
| Korean | 914 (97.86) | 4,733 (97.21) | 5,647 (97.31) | 0.002 |
| **Psychiatric Diseases** |  |  |  |  |
| No Disorders | 885 (0.95) | 4,706 (0.97) | 5,591 (96.35) | 0.007 |
| Anxiety Disorder | 8 (0.01) | 43 (0.01) | 51 (0.88) | 0.004 |
| Obsessive Disorder | 3 (0) | 8 (0) | 11 (0.19) | 0.281 |
| Trauma and Stress | 6 (0.01) | 26 (0.01) | 32 (0.55) | 0.077 |
| Psychotic Disorder | 11 (0.01) | 36 (0.01) | 47 (0.81) | 0.192 |
| Bipolar Disorder | 7 (0.01) | 13 (0) | 20 (0.34) | 0.451 |
| Depressive Disorder | 25 (0.03) | 87 (0.02) | 112 (1.93) | 0.166 |
| Personality Disorder | 0 (0) | 2 (0) | 2 (0.03) | 0.781 |
| Dissociative Disorder | 2 (0) | 20 (0) | 22 (0.38) | 0.205 |
| Somatoform Disorder | 2 (0) | 8 (0) | 10 (0.17) | 0.109 |

**Supplementary Table 3.** Characteristics of acute appendicitis-affected participants with and without complications (KUMC)

|  | With  Complication  (n=1,061) | Without Complication  (n=8,855) | Total  (n=9,916) | Standardized difference |
| --- | --- | --- | --- | --- |
|  | n (%) | n (%) | n (%) |  |
| **Sex** |  |  |  |  |
| Men | 548 (51.65) | 4,482 (50.62) | 5,030 (50.73) | 0.006 |
| **Age** |  |  |  |  |
| ≤ 14 | 146 (0.14) | 1,499 (0.17) | 1,645 (16.59) | 0.061 |
| 15ㅡ24 | 133 (0.13) | 1,889 (0.21) | 2,022 (20.39) | 0.147 |
| 25ㅡ34 | 139 (0.13) | 1,923 (0.22) | 2,062 (20.79) | 0.141 |
| 35 – 44 | 135 (0.13) | 1,236 (0.14) | 1,371 (13.83) | 0.028 |
| 45 – 54 | 131 (0.12) | 952 (0.11) | 1,083 (10.92) | 0.044 |
| 55 – 64 | 149 (0.14) | 712 (0.08) | 861 (8.68) | 0.191 |
| 65 – 74 | 125 (0.12) | 439 (0.05) | 564 (5.69) | 0.313 |
| ≥ 75 | 103 (0.1) | 205 (0.02) | 308 (3.11) | 0.57 |
| **Ethnicity** |  |  |  |  |
| Korean | 1,052 (99.15) | 8,827 (99.68) | 9,879 (99.63) | 0.002 |
| **Psychiatric Diseases** |  |  |  |  |
| No Disorders | 1,023 (0.96) | 8,718 (0.98) | 9,741 (98.24) | 0.006 |
| Anxiety Disorder | 6 (0.01) | 42 (0) | 48 (0.48) | 0.063 |
| Obsessive Disorder | 0 (0) | 1 (0) | 1 (0.01) | 1.412 |
| Trauma and Stress | 3 (0) | 17 (0) | 20 (0.2) | 0.135 |
| Psychotic Disorder | 4 (0) | 7 (0) | 11 (0.11) | 0.641 |
| Bipolar Disorder | 4 (0) | 14 (0) | 18 (0.18) | 0.321 |
| Depressive Disorder | 26 (0.02) | 65 (0.01) | 91 (0.92) | 0.468 |
| Personality Disorder | 0 (0) | 0 (0) | 0 (0) | 0.952 |
| Dissociative Disorder | 0 (0) | 1 (0) | 1 (0.01) | 1.412 |
| Somatoform Disorder | 3 (0) | 11 (0) | 14 (0.14) | 0.302 |

**Supplementary Table 4.** Characteristics of acute appendicitis-affected participants with and without complications (AUMC)

|  | With  Complication  (n=3,803) | Without Complication  (n=12,489) | Total  (n=16,292) | Standardized difference |
| --- | --- | --- | --- | --- |
|  | n (%) | n (%) | n (%) |  |
| **Sex** |  |  |  |  |
| Men | 2,072 (54.48) | 6,471 (51.81) | 8,543 (50.73) | 0.021 |
| **Age** |  |  | 0 (0) |  |
| ≤ 14 | 880 (0.23) | 2,710 (0.22) | 3,590 (16.59) | 0.027 |
| 15ㅡ24 | 446 (0.12) | 2,183 (0.17) | 2,629 (20.39) | 0.16 |
| 25ㅡ34 | 514 (0.14) | 2,796 (0.22) | 3,310 (20.79) | 0.198 |
| 35 – 44 | 600 (0.16) | 2,167 (0.17) | 2,767 (13.83) | 0.04 |
| 45 – 54 | 450 (0.12) | 1,168 (0.09) | 1,618 (10.92) | 0.103 |
| 55 – 64 | 349 (0.09) | 738 (0.06) | 1,087 (8.68) | 0.197 |
| 65 – 74 | 318 (0.08) | 441 (0.04) | 759 (5.69) | 0.404 |
| ≥ 75 | 246 (0.06) | 286 (0.02) | 532 (3.11) | 0.495 |
| **Ethnicity** |  |  | 0 (0) |  |
| Korean | 3,746 (98.5) | 12,349 (98.88) | 16,095 (99.63) | 0.002 |
| **Psychiatric Diseases** |  |  | 0 (0) |  |
| No Disorders | 3,751 (0.99) | 12,292 (0.98) | 16,043 (98.24) | 0.001 |
| Anxiety Disorder | 9 (0) | 53 (0) | 62 (0.48) | 0.228 |
| Obsessive Disorder | 1 (0) | 2 (0) | 3 (0.01) | 0.222 |
| Trauma and Stress | 11 (0) | 30 (0) | 41 (0.2) | 0.08 |
| Psychotic Disorder | 10 (0) | 16 (0) | 26 (0.11) | 0.331 |
| Bipolar Disorder | 1 (0) | 3 (0) | 4 (0.18) | 0.038 |
| Depressive Disorder | 19 (0) | 77 (0.01) | 96 (0.92) | 0.087 |
| Personality Disorder | 0 (0) | 6 (0) | 6 (0) | 0.038 |
| Dissociative Disorder | 0 (0) | 3 (0) | 3 (0.01) | 0.363 |
| Somatoform Disorder | 11 (0) | 50 (0) | 61 (0.14) | 0.132 |

**Supplementary Table 5.** Characteristics of acute appendicitis-affected participants with and without complications (EUMC)

|  | With  Complication  (n=2,360) | Without Complication  (n=13,147) | Total  (n=15,507) | Standardized difference |
| --- | --- | --- | --- | --- |
|  | n (%) | n (%) | n (%) |  |
| **Sex** |  |  |  |  |
| Men | 1,210 (51.27) | 6,315 (48.03) | 7,525 (50.73) | 0.024 |
| **Age** |  |  |  |  |
| ≤ 14 | 207 (0.09) | 3,195 (0.24) | 3,402 (16.59) | 0.299 |
| 15ㅡ24 | 302 (0.13) | 2,425 (0.18) | 2,727 (20.39) | 0.123 |
| 25ㅡ34 | 403 (0.17) | 2,802 (0.21) | 3,205 (20.79) | 0.077 |
| 35 – 44 | 429 (0.18) | 2,010 (0.15) | 2,439 (13.83) | 0.064 |
| 45 – 54 | 348 (0.15) | 1,280 (0.1) | 1,628 (10.92) | 0.16 |
| 55 – 64 | 306 (0.13) | 709 (0.05) | 1,015 (8.68) | 0.362 |
| 65 – 74 | 166 (0.07) | 385 (0.03) | 551 (5.69) | 0.362 |
| ≥ 75 | 186 (0.08) | 309 (0.02) | 495 (3.11) | 0.524 |
| **Ethnicity** |  |  |  |  |
| Korean | 2,316 (98.14) | 12,995 (98.84) | 15,311 (99.63) | 0.003 |
| **Psychiatric Diseases** |  |  |  |  |
| No Disorders | 2,313 (0.98) | 13,000 (0.99) | 15,313 (98.24) | 0.003 |
| Anxiety Disorder | 26 (0.01) | 70 (0.01) | 96 (0.48) | 0.297 |
| Obsessive Disorder | 0 (0) | 1 (0) | 1 (0.01) | 1.232 |
| Trauma and Stress | 7 (0) | 30 (0) | 37 (0.2) | 0.102 |
| Psychotic Disorder | 3 (0) | 2 (0) | 5 (0.11) | 1.046 |
| Bipolar Disorder | 2 (0) | 3 (0) | 5 (0.18) | 0.58 |
| Depressive Disorder | 16 (0.01) | 57 (0) | 73 (0.92) | 0.176 |
| Personality Disorder | 0 (0) | 4 (0) | 4 (0) | 0.435 |
| Dissociative Disorder | 1 (0) | 0 (0) | 1 (0.01) | 0.435 |
| Somatoform Disorder | 5 (0) | 19 (0) | 24 (0.14) | 0.15 |
